# Complete Genome Sequence of the First H5N1 Avian Influenza Virus Isolated from Chickens in Lebanon in 2016

**DOI:** 10.1128/genomeA.01062-16

**Published:** 2016-10-20

**Authors:** Elias Ibrahim, Abeer Sirawan, Bassel El-Bazzal, Jeanne El Hage, Mounir Abi Said, Hassan Zaraket, Ahmed Kandeil, Mohamed A. Ali, Ghazi Kayali

**Affiliations:** a Ministry of Agriculture, Beirut, Lebanon; b Lebanese Agricultural Research Institute, Animal Health Laboratory, Fanar, Lebanon; c Lebanese University, El Fanar, Lebanon; d American University of Beirut, Beirut, Lebanon; e Center of Scientific Excellence for Influenza Viruses, National Research Centre, Giza, Egypt; f Department of Epidemiology, Human Genetics, and Environmental Sciences, University of Texas Health Sciences Center, Houston, Texas, USA; g Human Link, Hazmieh, Lebanon

## Abstract

We generated the full genome of a highly pathogenic H5N1 avian influenza virus that caused an outbreak on a chicken farm in Lebnaon in April 2016. Analysis revealed that the virus belonged to clade 2.3.2.1c that recently caused outbreaks in West Africa and the United Arab Emirates.

## GENOME ANNOUNCEMENT

Highly pathogenic avian influenza (HPAI) H5N1 viruses were first detected in 1997 (1). Then, they spread widely and evolved rapidly into 10 distinct clades (0 to 9). Clades 2.1.3.2, 2.2.1.2, 2.3.2.1, 2.3.4.4, and 7.2 were detected in 2014 and continue to circulate globally (2). Clade 2.2 viruses were first detected in the Middle East in 2005 causing sporadic outbreaks in several countries and becoming enzootic in Egypt (3). No outbreaks were detected in Lebanon until April 2016, when a chicken farm in the Beqaa region had high mortality with symptoms resembling HPAI infection. The Lebanese Ministry of Agriculture detected HPAI H5 infection and successfully controlled the outbreak through culling and monitoring of neighboring farms.

An A/chicken/Lebanon/157/2016(H5N1) virus was isolated from infected chickens. Viral RNA was extracted using a QIAamp viral RNA minikit (Qiagen, Germany) according to the manufacturer’s protocol. The first-strand cDNA was synthesized using Superscript III reverse transcriptase (Invitrogen, CA) and Uni-12 primer (5′ AGCRAAAGCAGG3′) per manufacturer’s protocol. Genes were amplified using the Phusion master mix kit (Thermo, MA) and universal primers (4). Amplified products were sequenced at Macrogen (Macrogen, South Korea) and were assembled using SeqMan (DNASTAR, WI).

The eight genome segments encoded PB2, PB1, PB1-F2, PA, PA-X, HA, NP, NA, M1, M2, NS1, and NS2 viral proteins with lengths 759, 757, 57, 716, 252, 567, 498, 449, 252, 97, 225, and 121 amino acids (aa), respectively. The HA cleavage motif sequence of H5N1 isolates was RERRRKR*GLF, which is the signature of HPAI viruses. Analysis of the NXT/S motif (X can be any amino acid except proline) revealed that the isolate had seven potential glycosylation sites, at positions 10 (NNST), 23 (NVT), 140 (NSS), 165 (NNT), 286 (NSS), 483 (NGS), and 542 (NGS) (H5 numbering) within the HA molecule. The virus had Q222 and G224 at the receptor binding site, suggesting that it favors avian-like receptors (5). However, it also contained aa N and A at sites 94 and 133 instead of D and S, respectively, which is believed to enhance binding to α2-6 receptors (6). The analysis of the NA gene revealed a 20 aa deletion in the NA stalk region (positions 49 to 68). The virus had an NA substitution V149A that potentially reduced susceptibility to zanamivir (7). Mutations E627K and D701N in the PB2 that facilitate the adaptation of avian viruses to mammals, and increase transmission and/or pathogenicity (8, 9), were not recorded. The BLASTn and phylogenetic analysis of all eight segments revealed that the virus was closely related to the recent clade 2.3.2.1c viruses that circulated in West Africa (Ivory Coast, Niger, and Ghana) and India with high sequence homologies (98 to 99%). This clade has been previously detected in the United Arab Emirates in wild bird species (10). The emergence of this H5N1 clade in the Middle East is of both veterinary and human public health concern and requires surveillance at the human-animal interface.

### Accession number(s).

The complete genome sequence of the A/chicken/Lebanon/157/2016(H5N1) virus was deposited in GenBank under the accession numbers KX644138 to KX644145.
